# Haemophagocytic lymphohistiocytosis occurred during induction chemotherapy in an acute monocytic leukemia patient with FLT3-ITD and DNMT3A mutations

**DOI:** 10.1186/s12885-018-4534-z

**Published:** 2018-05-29

**Authors:** Fei Li, Xiaojie Zhang, Yunyun Wang, Ailin Yang, Zhanglin Zhang, Weiping Tang, Nan Zhong, Huidong Shi

**Affiliations:** 1 Department of Hematology, The First Affiliated Hospital of Nanchang University, 17 Yongwai Zheng Street, Nanchang, 330006 Jiangxi China; 2 Medical College, Nanchang University, Nanchang, 330006 China; 3 Georgia Cancer Center, Augusta University, Augusta, GA 30912 USA; 4 Department of Biochemistry and Molecular Biology, Medical College of Georgia, Augusta University, Augusta, GA 30912 USA; 5 Jiangxi Key Laboratory of Molecular Diagnosis and Precision Medicine, 17 Yongwai Zheng Street, Nanchang, 330006 Jiangxi China

**Keywords:** Haemophagocytic lymphohistiocytosis, Malignancy, Acute monocytic leukemia, FLT3-ITD, DNMT3A

## Abstract

**Background:**

Haemophagocytic lymphohistiocytosis (HLH) is considered to be a large challenge for clinicians due to the variable overlaps of symptoms with other severe diseases and a high rate of mortality. Prompt diagnosis and treatment are crucial to avoid a fatal outcome. However, very limited reports have focused on HLH during chemotherapy (Ch-HLH) due to a low incidence and insufficient knowledge.

**Case presentation:**

A 22-year-old male was diagnosed with acute monocytic leukemia with FLT3-ITD and DNMT3A mutations and pulmonary infection. He received IA regimen (Idarubicin, 8 mg/m2/d for 3 days and cytarabine, 100 mg/m2/d for 7 days) chemotherapy, anti-infection drugs and blood components transfusions. During the stage of bone marrow suppression, he presented with a fever, cytopenia (WBC, 0.43 × 10^9^/L; Hb, 73 g/L and PLT, 1 × 10^9^/L), refractory coagulation dysfunction (APTT, 104.0 s; PT, 30.5 s and Fbg, 0.87 g/L), splenomegaly (3 cm below the costal margin), hyperferritinemia (SF > 3000 μg/L), increased soluble interleukin-II receptors (sIL-2R > 7500 u/mL) and haemophagocytosis in the bone marrow and was diagnosed with HLH. After he was treated with methylprednisolone at 500 mg/d for 3 days, 120 mg/d for 3 days and 80 mg/d for 3 days, followed by a gradually reduced dose combined with powerful anti-infection drugs, his symptoms subsided and his abnormal parameters recovered to normal levels.

**Conclusion:**

Patients with HLH in acute leukemia have a high rate of mortality. This case report provides helpful clinical experiences relative to the recognition and treatment of Ch-HLH for clinicians.

## Background

Secondary haemophagocytic lymphohistiocytosis (HLH) is characterized by prolonged fever, hepatosplenomegaly, cytopenia, hypertriglyceridemia, hyperferritinemia and haemophagocytosis in the bone marrow, liver, spleen or lymph nodes. It is caused by a wide range of factors, including infections, malignancies, autoimmune diseases, metabolic diseases and acquired immune deficiencies. Of these, malignancy-associated HLH (M-HLH) is considered to be a large challenge to clinicians due to variable overlaps of symptoms with other types of HLH, sepsis and multiorgan failure, resulting in a higher incidence of misdiagnosis and mortality [1]. M-HLH can occur in newly diagnosed or relapsed malignancy (called “malignancy-triggered HLH”) and during chemotherapy, especially in the treatment course of leukemia or lymphoma (called “HLH during chemotherapy”, Ch-HLH). Prompt diagnosis and treatment are crucial to avoid a fatal outcome caused by multiorgan dysfunction. However, very few reports have focused on M-HLH, especially Ch-HLH, due to a low incidence and insufficient knowledge. Therefore, in this article, we present a rare case of HLH that occurred at the stage of induction chemotherapy in a patient with acute monocytic leukemia with FLT3-ITD and DNMT3A mutations.

## Case presentation

**Fig. 1:**
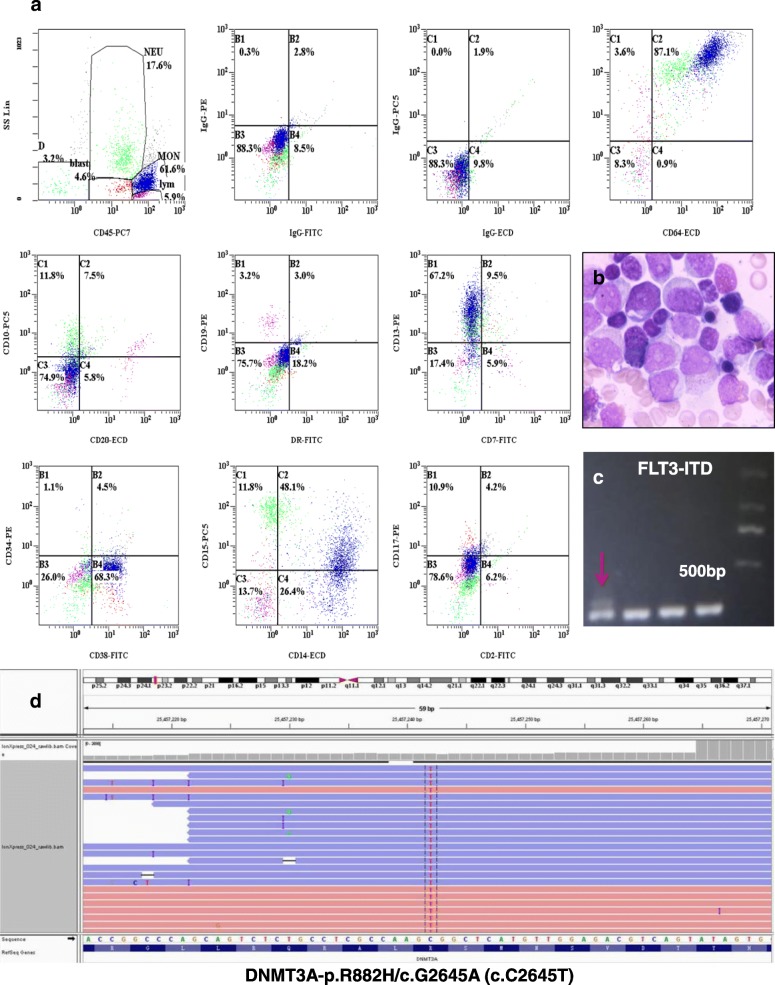
The patient was diagnosed with AML with FLT3-ITD and DNMT3A mutations. **a** Flow cytometry indicated that the leukemia cells positively expressed CD38, CD13, CD64, CD11b, CD15, CD14 and HLA-DR. **b** The bone marrow smear revealed AML. **c** FLT3-ITD Exon 11 mutation was found using PCR amplicon analyses. **d** DNMT3A Exon 23 c.G2645A mutations were found by next generation sequencing analyses using IGV software

A 22-year-old male was admitted to our centre due to a fever that lasted for 5 days (37.5–38.5 °C), coughing, expectoration, nose bleed and sporadic petechiae in both lower limbs. The physical examination indicated moist rales in his lung and sporadic petechiae in both lower limbs, but no enlarged superficial lymph nodes, liver or spleen. The blood cell count revealed 25.31 × 10^9^/L white blood cells (WBC) (normal range, 4-10 × 10^9^/L) with 30% blasts, 142 g/L haemoglobin (Hb) (normal range, 120–150 g/L), 11 × 10^9^/L platelets (PLT) (normal range, 100–300 × 10^9^/L), 30.6 g/L albumin (ALB) (normal range, 40–55 g/L), 303 U/L lactate dehydrogenase (LDH) (normal range, 0–248 U/L), 14 s prothrombin time (PT) (normal range, 9.8–12.1 s), 57.7 s activated partial prothrombin time (APTT) (normal range, 21.1–36.5 s), 18.0 s thrombin time (TT) (normal range, 14.0–21.0 s), 3.11 g/L fibrinogen (Fbg) (normal range, 1.8–3.5 g/L), 688 μg/L serum ferritin (SF) (normal range, 30–400 μg/L), 2.75 ng/mL procalcitonin (PCT) (< 0.5 ng/mL) and 44.5 mg/L C-reactive protein (CRP) (normal range, 0–8 mg/L). In addition, Epstein-Barr virus (EBV) and cytomegalovirus (CMV) DNA serological tests, blood culture (veins in both upper limbs, cultures for aerobic, anaerobic and fungal agents, three times), T-spot test, (1–3)-Beta-D-Glucan assay (G test) and galactomannan assay (GM test) were all negative. The counts for alanine aminotransferase (ALT), aspartate aminotransferase (AST), bilirubin (BIL), serum creatinine (Scr) and triglycerides (TG) were normal. The computed tomography (CT) scan examination revealed multiple high-density bilateral pulmonary parenchyma plaques, bilateral pleural effusion and a slightly enlarged spleen. The ECG showed sinus tachycardia. Echocardiography showed that the left ventricular ejection fraction was 65%. A bone marrow smear revealed acute monocytic leukemia with 43% blasts (Fig. 1b). Flow cytometry revealed tumour cells that positively expressed CD38, CD13, CD64, CD11b, CD15, CD14 and HLA-DR (Fig. 1a). The chromosome karyotype was normal. FLT3-ITD Exon 11 and DNMT3A Exon 23 c.G2645A mutations were detected (Fig. 1c and d), but no RUNX1-RUNX1T1, C-KIT/D816V, NPM1 or CEBPA mutations. Therefore, he was clinically diagnosed with acute monocytic leukemia with FLT3-ITD and DNMT3A mutations and pulmonary infection. He received IA regimen chemotherapy (Idarubicin, 8 mg/m^2^/d, for 3 days and cytarabine, 100 mg/m^2^/d, for 7 days), anti-infection drug treatments (Imipenem/cilastatin, vancomycin hydrochloride and voriconazole, time from admission to d6 during the stage of IA chemotherapy) and blood components transfusions.

**Fig. 2:**
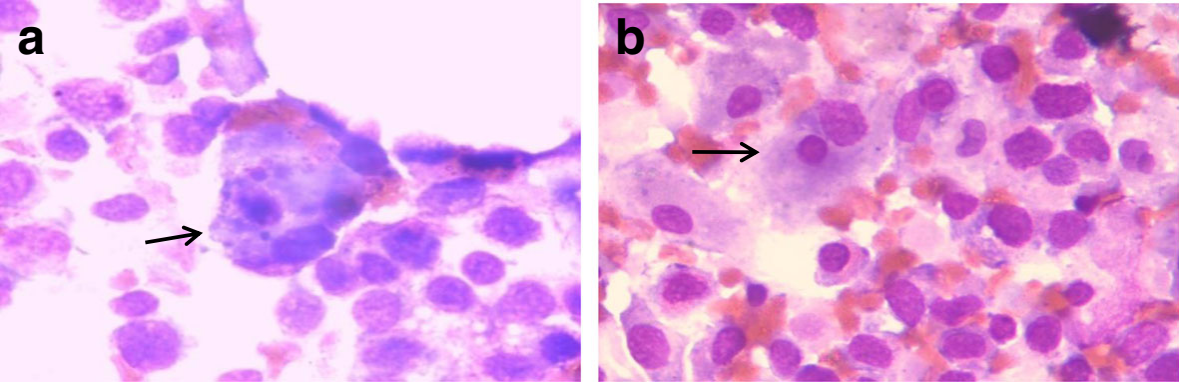
The bone marrow cell morphology of the patient at 14 days after finishing IA chemotherapy. **a** and **b** indicate haemophagocytic cells (at the arrowhead) and some promonocytes can be seen in the bone marrow smear (Wright-Giemsa staining, × 1,000)

**Fig. 3:**
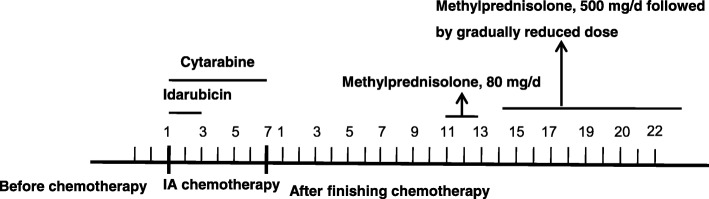
The treatment course of the patient (each small grid represents 1 day)

In the following days, his lung infection gradually improved and his body temperature decreased to the normal level. SF, PCT and CRP decreased to 324 μg/L, 0.75 ng/mL and 25.4 mg/L, respectively. Multiple high-density bilateral pulmonary parenchyma plaques were obviously absorbed. However, due to bone marrow suppression after chemotherapy, he developed a fever again, his body temperature was increased to over 39 °C and his blood cell counts decreased to 0.43 × 10^9^/L WBC, 73 g/L Hb and 1 × 10^9^/L PLT in the 11 days after finishing IA chemotherapy. To our surprise, his coagulation dysfunction worsened, with APTT and PT prolonged to 104.0 s and 30.5 s, respectively, and Fbg decreased to 0.87 g/L. TT and the plasma protamine paracoagulation test (3P test) were normal. PCT and CRP increased to 3.8 ng/mL and 35.7 mg/L again, respectively. Serological tests for EBV-DNA and CMV-DNA, a blood culture, the G test and the GM test were still negative. The levels of ALT, AST, BIL, Scr and TG were normal. The patient was originally diagnosed with severe infection, systemic inflammatory response syndrome (SIRS), and disseminated intravascular coagulation (DIC) and was treated with 80 mg/d methylprednisolone for 3 days (from d11 to d13 after finishing chemotherapy), fresh frozen plasma, platelets, cryoprecipitate anti-fibrinolysis drugs, and powerful antibiotics, including cefoperazone/sulbactam, tigecycline and voriconazole (from d10 after finishing chemotherapy until his temperature returned to the normal level and granulocytic deficiency resolved). However, his coagulation dysfunction did not improve and his peripheral blood cells were not restored at 14 days after finishing IA chemotherapy following the above treatments. He was subjected to bone marrow aspiration. Surprisingly, cell proliferation in his bone marrow was extremely active, with 7% blasts and 5% haemophagocytic cells (Fig. 2a and b). In addition, his SF was > 3000 μg/L, soluble interleukin-II receptor (sIL-2R) was > 7500 u/mL, and spleen was 3 cm below the left costal margin. Table 1 shows the patient’s clinical parameters. Based on the fever, hypofibrinogenemia, splenomegaly, cytopenia, hyperferritinemia, increased sIL-2R and haemophagocytosis in bone marrow, he was diagnosed with HLH. Because of incomplete cytokine storms blockade, the previous dose of methylprednisolone presumably did not effectively suppress HLH. Therefore, he was given 500 mg/d of methylprednisolone for 3 days (d14–16 after finishing chemotherapy), 120 mg/d for d17–19, and 80 mg/d for d20–22, followed by a gradually reduced dose. Eventually, his temperature dropped to the normal level, his coagulation dysfunction gradually improved, with an APTT of 46.2 s, PT of 11.8 s and Fbg of 4.82 g/L, and his peripheral blood cells were restored to 15.0 × 10^9^/L WBC, 78 g/L Hb and 42 × 10^9^/L PLT. However, 34% of blasts were still observed in the bone marrow smear at 20 days after finishing IA chemotherapy. The patient is still alive and being followed. Figure 3 shows the treatment course of the patient.

**Table 1:** The clinical parameters of the patient

Parameters	Before chemotherapy	d6 during the IA chemotherapy	d11–14 after finishing chemotherapy	d20 after finishing chemotherapy
WBC (×10^9^/L)	25.3	3.2	0.4	15.0
Hb (g/L)	142	66	73	78
PLT (×10^9^/L)	11.0	5	1	42
APTT (s)	57.7	72	104	46.2
PT (s)	14.0	14.6	30.5	11.8
Fbg (g/L)	3.1	2.2	0.87	4.8
TG (mmol/L)	1.5	–	0.8	0.9
SF (μg/L)	688	324	> 3000	1259
PCT (ng/mL)	2.8	0.75	3.8	0.7
CRP (mg/L)	44.5	25.4	35.7	49.1
LDH (U/L)	303	481	57	392

*WBC* white blood cell (normal range, 4-10 × 10^9^/L), *Hb* hemoglobin (normal range, 120–150 g/L), *PLT* platelet (normal range,100–300 × 10^9^/L), *APTT* activated partial prothrombin time (normal range, 21.1–36.5 s), *P T* prothrombin time (normal range, 9.8–12.1 s), *Fbg* fibrinogen (normal range, 1.8–3.5 g/L), *TG* triglycerides (normal range, 0–1.7 mmol/L), *SF* serum ferritin (normal range, 30–400 μg/L), *PCT*:procalcitonin (< 0.5 ng/mL), *CRP* C-reactive protein (normal range, 0–8 mg/L), *LDH* lactate dehydrogenase (normal range, 0–248 U/L), *IA regimen* idarubicin, 8 mg/m^2^/d at d1–3, Cytarabine, 100 mg/m^2^/d at d1–7

## Discussion

In recent years, M-HLH with a high rate of misdiagnosis and mortality has gained increasing attention from clinicians because its symptoms are nonspecific and overlap with some severe illnesses, including sepsis, SIRS and multiorgan failure. The most common tumour types that trigger HLH are haematological neoplasms (93.7%) with T-cell or natural-killer (NK) lymphoma or leukemia (35.2%), followed by B-cell lymphoma (31.8%), other non-specified hematologic neoplasms (14.4%) and Hodgkin lymphoma (5.8%). Of these, leukemia-triggered HLH accounted for only 6.4% of cases [2–4]. According to consensus recommendations for the diagnosis and management of HLH-associated malignancies, M-HLH can occur in the phase of diagnosis or chemotherapy, including induction, consolidation, and even maintenance [2, 5].

So far, very few sporadic reports on M-HLH during the onset of acute myeloid leukemia (AML) or course of chemotherapy have been reported [6–10]. Excessive cytokines secreted by malignant cells and/or viruses may be the cause of AML-triggered HLH, and viruses, invasive fungi and bacterial infections after chemotherapy may be the major triggers of Ch-HLH. In the present case, the patient had a fever, hypofibrinogenemia, splenomegaly, cytopenia, hyperferritinemia, increased sIL-2R levels and haemophagocytosis in the bone marrow during the induction of chemotherapy, fulfilling the diagnostic criteria of HLH 2004 [11]. Lehmberg et al. [10] reported 21 cases of M-HLH. Among these, 8 patients had Ch-HLH, including 2 cases of AML, 5 cases of acute lymphoblastic leukemia (ALL) and 1 case of lymphoma; 6 patients occurred in the stage of remission; and 2 patients occurred in the stage of induction chemotherapy. In addition, *E. coli*, EBV, HHV6, aspergillus, adenovirus and CMV were found in the seven patients. Although we actively sought infectious causes, including EBV, CMV, and herpes simplex virus, and performed a blood culture, a G test and a GM test, the positive triggers remained unclear. It is possible that HLH was co-triggered by excessive cytokine secretion by leukemia cells and lung infection in this patient.

Patients with M-HLH and Ch-HLH show very poor survival, with a median overall survival of 0.9–1.2 years and 6-month survival rates of 67 and 63%, respectively [2, 10]. This patient had FLT3-ITD Exon 11 and DNMT3A Exon 23 c.G2645A mutations, which predict a worse prognosis. In fact, the patient was refractory to chemotherapy and achieved no remission after a cycle of standard regimen chemotherapy. The relationship between HLH and some subtypes of leukemia such as FLT3-ITD and/or DNMT3A mutations is unclear.

The best therapeutic approach for Ch-HLH remains elusive. Based on iatrogenic immunosuppression and consecutive triggering infections, if infections are detected in patients with Ch-HLH, the consensus is that chemotherapy should be postponed and powerful anti-infection treatment should be considered first [2]. In the present case, the patient was given 80 mg/d methylprednisolone for 3 days and fresh frozen plasma, platelets, cryoprecipitate, anti-fibrinolysis drugs and powerful antibiotics to control the inflammation response and his coagulation dysfunction. However, his abnormal parameters did not improve or become restored. Due to an uncontrolled hyperinflammation response, he was then treated with methylprednisolone at 500 mg/d for 3 days and 120 mg/d for 3 days, followed by a gradually reduced dose. His temperature and coagulation dysfunction were then successfully controlled to normal levels, suggesting that a sufficient dose of glucocorticosteroids may be effective to control the hyperinflammation response in Ch-HLH patients.

## Conclusions

In summary, Ch-HLH is a life-threatening disease with very high mortality. Early recognition, a sufficient dose of glucocorticosteroids and regulation of the hyperinflammation response are crucial to avoid a fatal outcome due to multiorgan dysfunction and improve the overall survival of these patients. The present case report may provide some clinical experiences regarding the recognition and treatment of Ch-HLH for clinicians.

## Abbreviations

3P test: plasma protamine paracoagulation test
ALB: Albumin
ALL: Acute lymphoblastic leukemia
ALT: Alanine aminotransferase
AML: Acute myeloid leukemia
APTT: Activated partial prothrombin time
AST: Aspartate aminotransferase
BIL: Bilirubin
CMV: Cytomegalovirus
CRP: C-reactive protein
CT: Computed tomography
DIC: Disseminated intravascular coagulation
EBV: Epstein-Barr virus
Fbg: Fibrinogen
G test: (1–3)-Beta-D-Glucan Assay
GM test: Galactomannan assay
Hb: Haemoglobin
HLH: Secondary haemophagocytic lymphohistiocytosis
IA regimen: Idarubicin and cytarabine
LDH: Lactate dehydrogenase
M-HLH: Malignancy-associated HLH
PCT: Procalcitonin
PLT: Platelets
PT: Prothrombin time
Scr: Serum creatinine
SF: Serum ferritin
sIL-2R: Soluble interleukin-II receptor
SIRS: Systemic inflammatory response syndrome
TG: Triglycerides
TT: Thrombin time
WBC: White blood cell
